# Plasma proteomic profile of frailty

**DOI:** 10.1111/acel.13193

**Published:** 2020-08-06

**Authors:** Sanish Sathyan, Emmeline Ayers, Tina Gao, Sofiya Milman, Nir Barzilai, Joe Verghese

**Affiliations:** ^1^ Department of Neurology Albert Einstein College of Medicine Bronx NY USA; ^2^ Institute for Aging Research, Department of Medicine Albert Einstein College of Medicine Bronx NY USA; ^3^ Department of Genetics Albert Einstein College of Medicine Bronx NY USA

**Keywords:** aging, cumulative frailty score, frailty, frailty prediction, proteomics, SomaScan^®^ assay

## Abstract

Frailty is a state of decreased physiological reserve and increased vulnerability to adverse outcomes in aging, and is characterized by dysregulation across various biological pathways. Frailty may manifest biologically as alteration in protein expression, possibly regulated at genetic, transcriptional and epigenetic levels. In this study, we examined the proteomic profile associated with frailty defined by an established cumulative frailty index (FI). Using the SomaScan^®^ assay, 4265 proteins were measured in plasma, of which 55 were positively associated and 88 were negatively associated with the FI. The proteins most strongly associated with frailty were fatty acid‐binding proteins, including fatty acid‐binding protein (FABP) (*p* = 1.96 × 10^−19^) and FABPA (*p* = 8.10 × 10^−16^), leptin (*p* = 1.43 × 10^−14^), and ANTR2 (*p* = 7.95 × 10^−20^). Pathway analysis with the top 143 frailty‐associated proteins revealed enrichment for proteins in pathways related to lipid metabolism, musculoskeletal development and function, cell‐to‐cell signaling and interaction, cellular assembly, and organization. Frailty prediction model constructed with elastic net regression utilizing 110 proteins demonstrated a correlation between predicted frailty and observed frailty (*r* = 0.57, *p* < 2.2 × 10^−16^). Predicted frailty was also more strongly correlated with chronological age (*r* = 0.54, *p* < 2.2 × 10^−16^) than observed frailty (*r* = 0.37, *p* = 1.2 × 10^−15^). This study identified novel proteins and pathways related to frailty that may offer improved frailty phenotyping and prediction.

## Graphical abstract

Proteomic changes associated with frailty were studied using SOMAScan proteomic assay analyzing ~4000 proteins identifying novel proteins and pathways related to frailty that may offer improved frailty phenotyping and prediction.

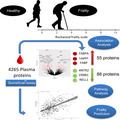

## 1 INTRODUCTION

Frailty is a late life phenotype, which is associated with low physiologic reserve and increased vulnerability to adverse outcomes such as disability, hospitalization, and death (Fried, Darer, & Walston, 2003; Fried, Ferrucci, Darer, Williamson, & Anderson, 2004). Frailty is a multidimensional construct and involves several components, including physical, psychological, cognitive, and social domains (Fried et al., 2001; Gobbens, van Assen, Luijkx, & Schols, 2012). The complexity of this clinical syndrome has made it difficult to elucidate its biology. Although both genetic and proteomic approaches have been applied, previous studies have been inconclusive regarding the biology of frailty. The main limitations in previous proteomic studies were the fewer number of proteins analyzed as well as the small sample sizes. A study that compared six frail to six non‐frail older adults found 31 out of the 226 proteins examined to be highly expressed in frail participants compared to non‐frail participants including angiotensinogen (ANGT), kininogen‐1 (KG), and antithrombin III (AT) (Lin et al., 2017). Another small study that focused on glycoproteins identified an association of haptoglobin, transferrin, and fibrinogen with frailty (Shamsi et al., 2012). A number of studies targeting candidate proteins in pathways related to oxidative stress and inflammation revealed an association between frailty with Interleukin‐6 and lipoprotein phospholipase A2 (Ershler & Keller, 2000; Liu et al., 2016). However, only limited conclusions can be drawn from these prior studies in relationship to the biology of frailty as they were based on a candidate pathway approaches and were restricted to few participants as well as proteins. To date, no large‐scale proteomic study has been carried out in regard to frailty. An additional challenge is to distinguish the biological antecedents of frailty from aging. Since frailty is strongly associated with chronological age, both may share a common biological signature (Xue, 2011).

Frailty is a multidimensional concept that stems from imbalance in multiple biological pathways. Thus, this complex clinical phenotype may be better interrogated by employing an unbiased approach focused on high‐throughput proteomic or genomic analysis. To elucidate the proteomic signature associated with frailty, we examined the cross‐sectional association between 4265 proteins and frailty in 880 community‐residing Ashkenazi Jewish (AJ) older adults participating in the LonGenity Study (Lehallier et al., 2019; Sathyan et al., 2018). We employed an unbiased approach using the SomaScan assay, which is a highly multiplexed, sensitive, quantitative, and reproducible proteomic tool that can assess thousands of proteins simultaneously in a single blood sample (Candia et al., 2017). The principle of SOMAmer^®^ reagents is based on aptamer technology that uses single stranded DNA‐based protein affinity reagents. We defined frailty using the established cumulative frailty index (FI) proposed by Rockwood et al. that includes a diverse range of deficits to capture the complex and multidimensional nature of the frailty phenotype (Searle, Mitnitski, Gahbauer, Gill, & Rockwood, 2008). Further, we developed a model to predict frailty based on proteomic markers. Establishing the proteomic signature of frailty using an unbiased approach and a comprehensive frailty definition may provide new insights into pathways and underlying biology of frailty in aging.

## 2 RESULTS

### 2.1 Study population

Among the 880 eligible individuals in the LonGenity cohort who had both phenotypic and proteomic data available, 448 were offspring of parents with usual survival (OPUS) and 432 were offspring of parents with exceptional longevity (OPEL). Demographic and clinical characteristics are summarized in Table 1. The mean FI score for the eligible study sample was 0.163 (standard deviation [*SD*] = 0.086). The mean frailty scores for OPEL and OPUS were 0.151 ± 0.079 and 0.175 ± 0.091, respectively. The mean age of the participants was 75.35 ± 6.56 years, and 54.8% were women. The sample was highly educated with mean years of education being 17.52 ± 2.88 years.

**Table 1:** Clinical characteristics of cohort.

Variables	LonGenity	OPEL	OPUS
Participants	880	432 (49.1%)	448 (50.9%)
Age, mean ± *SD*, years	75.35 ± 6.56	74.35 ± 6.00	76.31 ± 6.92
Women, %	482 (54.8)	258 (59.7%)	224 (50%)
Education, mean, years	17.52 ± 2.88	17.69 ± 2.93	17.35 ± 2.81
Rockwood frailty Score (Mean ± *SD*)	0.163 ± 0.086	0.151 ± 0.079	0.175 ± 0.091
Medical illnesses			
Stroke, %	3.3	1.4	5.2
Diabetes, %	8.5	6.9	10.0
Myocardial infarction,%	5.8	4.9	6.7
Arthritis, %	41.6	41.7	41.6
Hypertension, %	43.5	37.3	49.6

### 2.2 Association analysis with frailty

**Figure 1:**
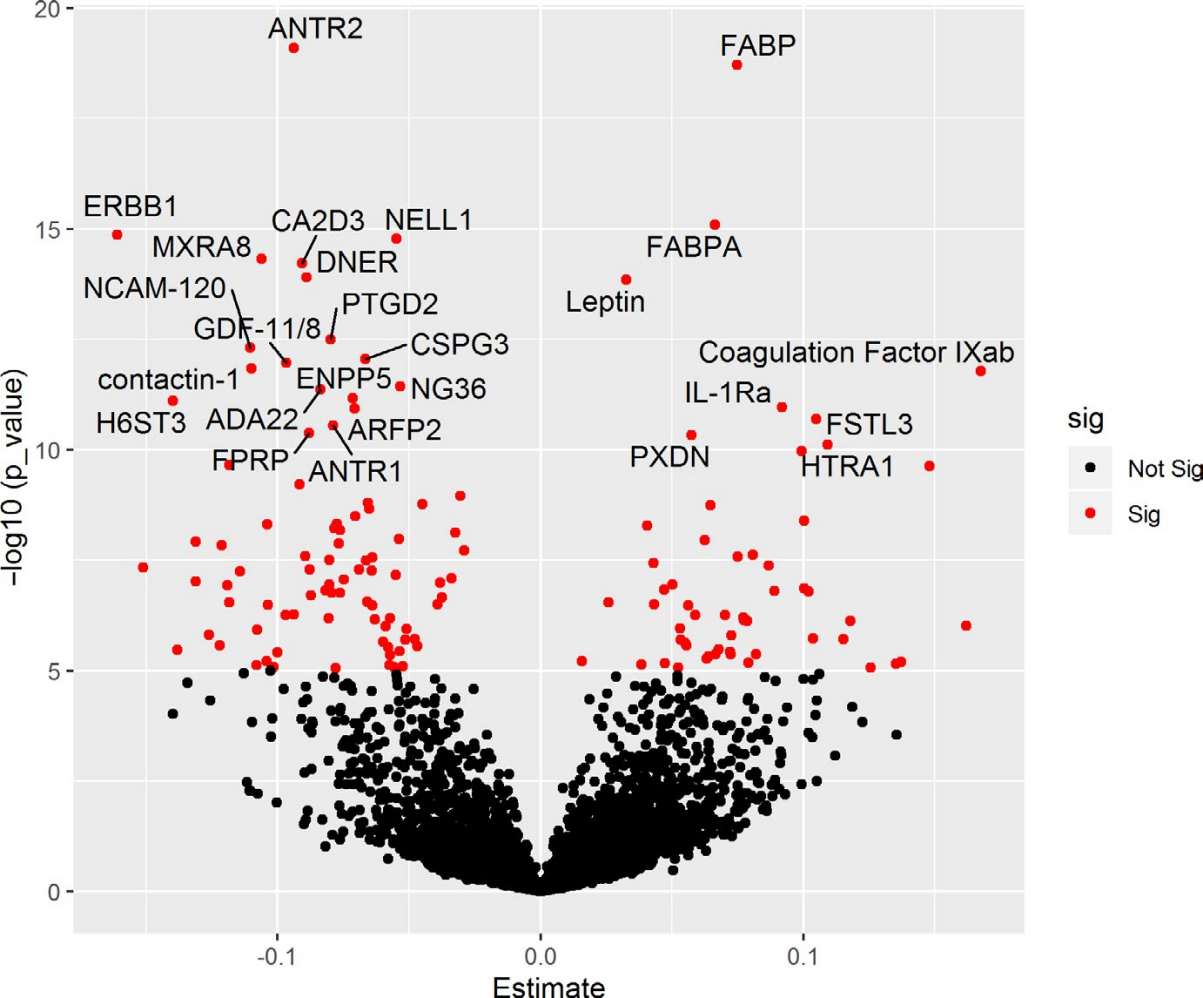
Association of proteins with frailty phenotype. Volcano plot showing associated proteins as red dots (*p*‐value < 1.0 × 10^−5^) X‐axis denotes the Beta estimates coefficient from linear model and Y‐axis shows the significance level presented as –log_10_ (*p*‐value). Top proteins have been annotated

There were 143 proteins that were significantly associated with the cumulative FI (Figure 1). Of these, 55 proteins were positively associated with the FI, while 88 proteins were negatively associated with the FI.

#### 2.2.1 Increased expression of proteins with frailty

The top two proteins associated positively with FI were fatty acid‐binding protein, heart (FABP) (*β* [*SE*] =0.0748 [0.0081]; *p* = 1.96 × 10^−19^), and fatty acid‐binding protein, adipocyte (FABPA) (*β* [*SE*] =0.0665 [0.0081]; *p* = 8.10 × 10^−16^) (Figure 1). Other proteins whose expression was increased with frailty included leptin (*β* [*SE*] =0.0327 [0.0042]; *p* = 1.43 × 10^−14^), coagulation factor IXab (*β* [*SE*] =0.1676 [0.0234]; *p* = 1.64 × 10^−12^), interleukin‐1 receptor antagonist protein (IL‐1Ra) (*β* [*SE*] =0.0918 [0.0133]; *p* = 1.08 × 10^−11^), follistatin‐related protein 3 (FSTL3) (*β* [*SE*] =0.1048 [0.0154]; *p* = 2.01 × 10^−11^), peroxidasin homolog (PXDN) (*β* [*SE*] =0.0574 [0.0086]; *p* = 4.66 × 10^−11^), and HtrA serine peptidase 1 (HTRA1) (*β* [*SE*] =0.1093 [0.0166]; *p* = 7.55 × 10^−11^). The proteins that were the most strongly positively associated with the FI are shown in Table 2.

**Table 2:** Top 30 most significant SOMAmer reagent targets associated positively (increased expression) with frailty phenotype

SeqId	SomaId	UniProt	Target	Target full name	Estimate	Std Error	*p*‐value
5437‐63	SL001774	P05413	FABP	Fatty acid‐binding protein, heart	0.0748	0.0081	1.96E−19
15386‐7	SL005086	P15090	FABPA	Fatty acid‐binding protein, adipocyte	0.0665	0.0081	8.10E−16
8484‐24	SL000498	P41159	Leptin	Leptin	0.0327	0.0042	1.43E−14
5307‐12	SL004400	P00740	Coagulation Factor IXab	Coagulation factor IXab	0.1676	0.0234	1.64E−12
5353‐89	SL001990	P18510	IL‐1Ra	Interleukin‐1 receptor antagonist protein	0.0918	0.0133	1.08E−11
3438‐10	SL009324	O95633	FSTL3	Follistatin‐related protein 3	0.1048	0.0154	2.01E−11
13463‐1	SL007573	Q92626	PXDN	Peroxidasin homolog	0.0574	0.0086	4.66E−11
15594‐47	SL008268	Q92743	HTRA1	Serine protease HTRA1	0.1093	0.0166	7.55E−11
12987‐12	SL019472	Q16629	SRSF7	Serine/arginine‐rich splicing factor 7	0.0994	0.0152	1.08E−10
4876‐32	SL000357	P00740	Coagulation Factor IX	Coagulation factor IX	0.1480	0.0231	2.36E−10
11214‐40	SL019363	Q9UBS3	DNJB9	DnaJ homolog subfamily B member 9	0.0646	0.0106	1.85E−09
10702‐1	SL012521	Q2UY09	COSA1	Collagen alpha‐1(XXVIII) chain	0.1004	0.0169	4.05E−09
7211‐2	SL005355	P07998	RNase 1	Ribonuclease pancreatic	0.0405	0.0069	5.18E−09
19233‐75	SL006960	O00244	ATOX1	Copper transport protein ATOX1	0.0626	0.0109	1.13E−08
12574‐36	SL000405	P20800	Endothelin 2	Endothelin‐2	0.0807	0.0143	2.43E−08
17706‐4	SL020934	Q13522	PPR1A	Protein phosphatase 1 regulatory subunit 1A	0.0749	0.0133	2.65E−08
4968‐50	SL008099	P40121	CAPG	Macrophage‐capping protein	0.0431	0.0078	3.68E−08
15441‐6	SL008548	P17900	SAP3	Ganglioside GM2 activator	0.0869	0.0157	4.20E−08
3339‐33	SL007206	P35442	TSP2	Thrombospondin‐2	0.0503	0.0094	1.13E−07
5644‐60	SL007198	P34096	RNAS4	Ribonuclease 4	0.1003	0.0189	1.37E−07
7638‐30	SL005403	Q12907	Lectin, mannose‐binding 2	Vesicular integral‐membrane protein VIP36	0.0471	0.0089	1.45E−07
11196‐31	SL004928	P12111	Collagen alpha‐3(VI)	Collagen alpha‐3(VI) chain	0.0890	0.0168	1.58E−07
9366‐54	SL004847	Q9HBE5	IL‐21 sR	Interleukin‐21 receptor	0.1020	0.0193	1.62E−07
19335‐2	SL005988	Q9UK76	HN1	Hematological and neurological expressed 1 protein	0.0260	0.0050	2.88E−07
4374‐45	SL003869	Q99988	MIC‐1	Growth/differentiation factor 15	0.0432	0.0084	3.20E−07
12373‐73	SL014875	P62995	TRA2B	Transformer‐2 protein homolog beta	0.0562	0.0109	3.40E−07
16292‐288	SL011908	P09681	GIP	Gastric inhibitory polypeptide	0.0703	0.0139	5.54E−07
19241‐31	SL005353	P82980	RBP‐III	Retinol‐binding protein 5	0.0589	0.0117	5.61E−07
6379‐62	SL012648	Q86TH1	ATL2	ADAMTS‐like protein 2	0.0772	0.0154	6.27E−07
11590‐5	SL019537	Q86U06	RBM23	Probable RNA‐binding protein 23	0.0774	0.0155	7.25E−07

#### 2.2.2 Decreased expression of proteins with frailty

Top proteins that were negatively associated with FI were anthrax toxin receptor 2(ANTR2) (*β* [*SE*] = −0.0938 [0.0100]; *p* = 7.95 × 10^−20^), epidermal growth factor receptor (ERBB1) (*β* [*SE*] = −0.1611 [0.0198]; *p* = 1.38 × 10^−15^), and neural EGF Like‐Like molecule 1(NELL1) (*β* [*SE*] = −0.0547 [0.0067]; *p* = 1.66 × 10^−15^) (Figure 1). The proteins with the most negative associations with frailty are listed in Table 3.

**Table 3:** Top 30 most significant SOMAmer reagent targets associated negatively (decreased expression) with frailty phenotype

SeqId	SomaId	UniProt	Target	Target full name	Estimate	Std Error	*p*_value
15559‐5	SL011048	P58335	ANTR2	Anthrax toxin receptor 2	−0.0938	0.0100	7.95E−20
2677‐1	SL002644	P00533	ERBB1	Epidermal growth factor receptor	−0.1611	0.0198	1.38E−15
6544‐33	SL012542	Q92832	NELL1	Protein kinase C‐binding protein NELL1	−0.0547	0.0067	1.66E−15
10521‐10	SL017989	Q9BRK3	MXRA8	Matrix‐remodeling‐associated protein 8	−0.1061	0.0133	4.80E−15
8885‐6	SL018710	Q8IZS8	CA2D3	Voltage‐dependent calcium channel subunit alpha‐2/delta‐3	−0.0908	0.0114	6.09E−15
9769‐48	SL008968	Q8NFT8	DNER	Delta and Notch‐like epidermal growth factor‐related receptor	−0.0891	0.0113	1.23E−14
12549‐33	SL014636	O60760	PTGD2	Hematopoietic prostaglandin D synthase	−0.0798	0.0108	3.21E−13
4498‐62	SL003764	P13591	NCAM‐120	Neural cell adhesion molecule 1, 120 kDa isoform	−0.1106	0.0151	4.89E−13
15573‐110	SL008782	O14594	CSPG3	Neurocan core protein	−0.0667	0.0092	8.88E−13
2765‐4	SL021043	O95390 O14793	GDF‐11/8	Growth/differentiation factor 11/8	−0.0967	0.0134	1.08E−12
2974‐61	SL004855	Q12860	contactin‐1	Contactin‐1	−0.1100	0.0153	1.45E−12
5843‐60	SL003542	Q96KQ7	NG36	Histone‐lysine N‐methyltransferase EHMT2	−0.0534	0.0076	3.66E−12
7933‐75	SL018350	Q9P0K1	ADA22	Disintegrin and metalloproteinase domain‐containing protein 22	−0.0838	0.0119	4.30E−12
6556‐5	SL012863	Q9UJA9	ENPP5	Ectonucleotide pyrophosphatase/phosphodiesterase family member 5	−0.0713	0.0102	6.83E−12
18896‐23	SL021212	Q8IZP7	H6ST3	Heparan‐sulfate 6‐O‐sulfotransferase 3	−0.1397	0.0201	7.92E−12
12630‐8	SL019787	P53365	ARFP2	Arfaptin‐2	−0.0706	0.0103	1.16E−11
10464‐6	SL008696	Q9H6X2	ANTR1	Anthrax toxin receptor 1	−0.0788	0.0117	2.87E−11
12727‐7	SL019877	Q9P2B2	FPRP	Prostaglandin F2 receptor negative regulator	−0.0881	0.0132	4.24E−11
8900‐28	SL007582	Q92859	NEO1	Neogenin	−0.1184	0.0184	2.17E−10
5698‐60	SL005005	P22105	Tenascin‐X	Tenascin‐X	−0.0916	0.0146	6.15E−10
4187‐49	SL000247	P52209	6‐Phosphogluconate dehydrogenase	6‐phosphogluconate dehydrogenase, decarboxylating	−0.0304	0.0049	1.12E−09
3235‐50	SL010391	Q8TEU8	WFKN2	WAP, Kazal, immunoglobulin, Kunitz and NTR domain‐containing protein 2	−0.0656	0.0108	1.60E−09
7210‐25	SL004470	P51693	Amyloid‐like protein 1	Amyloid‐like protein 1	−0.0450	0.0074	1.73E−09
8841‐65	SL008847	Q8IUL8	CILP2	Cartilage intermediate layer protein 2	−0.0651	0.0107	2.13E−09
6388‐21	SL017451	Q96EE4	CC126	Coiled‐coil domain‐containing protein 126	−0.0706	0.0118	3.22E−09
15491‐20	SL008437	Q9HCU0	CD248	Endosialin	−0.0775	0.0131	4.72E−09
10833‐64	SL012788	Q96QV1	HHIP	Hedgehog‐interacting protein	−0.1040	0.0176	4.97E−09
8039‐41	SL018368	Q8N128	F177A	Protein FAM177A1	−0.0783	0.0133	5.92E−09
6440‐31	SL012493	Q13361	MFAP5	Microfibrillar‐associated protein 5	−0.0763	0.0130	6.58E−09
10565‐19	SL017951	O94933	SLIK3	SLIT and NTRK‐like protein 3	−0.0323	0.0055	7.64E−09

Additionally, the analysis revealed significant associations between FI and proteins that previously have been linked with aging, like c‐reactive protein (CRP) (*β* [*SE*] =0.0158 [0.0035]; *p* = 6.19 × 10^−6^) and KLOTHO (*β* [*SE*] = −0.0538 [0.0093]; *p* = 1.04 × 10^−8^). We also found a significant association of GDF‐15 protein with frailty (*β* [*SE*] =0.0432 [0.0084; *p* = 3.20 × 10^−7^).

*Gender stratified analysis* showed a significant difference in the number of proteins associated with frailty in male and female participants. There were 88 proteins associated with frailty in the 482 female participants, while only 16 proteins were associated with frailty in 398 male participants (Table S1 and S2; Figure S1). Top hit proteins, including FABP, ANTR2, NELL1, and FABPA, were associated with frailty in both genders (Tables S1 and S2). Proteins that were significantly associated only in males include BCMA (B‐cell maturation antigen alias tumor necrosis factor receptor superfamily member 17), TSP2 (thrombospondin‐2), contactin‐4, F177A (family with sequence similarity 177 member A1), CD248 (endosialin), and EFS (embryonal Fyn‐associated substrate) (Table S1).

### 2.3 Pathway analysis

Pathway analysis using IPA revealed that proteins related to lipid metabolism were the top "*molecular and cellular functions"* associated with frailty (Table S3). This was supported by top associated proteins FABP, FABPA, and leptin, which have known roles in lipid metabolism. *Network analysis* in IPA showed “Organ Morphology, Skeletal and Muscular System Development and Function, Cell Morphology” to be the top network associated with the frailty phenotype (Table S4). The second network associated with frailty was related to tissue development and cell signaling, while the third network was involved in lipid metabolism (Table S4). The results obtained in IPA were also validated with other pathway analyses tools. Reactome pathway analysis pointed toward extracellular matrix organization and glycosaminoglycan metabolism as top pathways associated with frailty. STRING analysis showed enrichment of cell adhesion, system development, and multicellular organismal process as top biological process associated with frailty.

Post hoc analysis was performed with the top lipid metabolism‐associated proteins (FABP, FABPA, and leptin) to further understand their role in frailty. We adjusted the primary model for clinical (body mass index [BMI]) and biological (total cholesterol level) markers of lipid metabolism. The strength of association of FABP (*β* [*SE*] =0.0479 [0.0095]; *p* = 6.44 × 10^−07^) and FABPA (*β* [*SE*] =0.0471 [0.0089]; *p* = 1.67 × 10^−7^) with frailty was weaker but still significant in this exploratory model. Leptin (*β* [*SE*] =0.0102 [0.0059]; *p* = 0.0832) was no longer significantly associated with frailty.

### 2.4 Frailty prediction using proteomic markers

**Figure 2:**
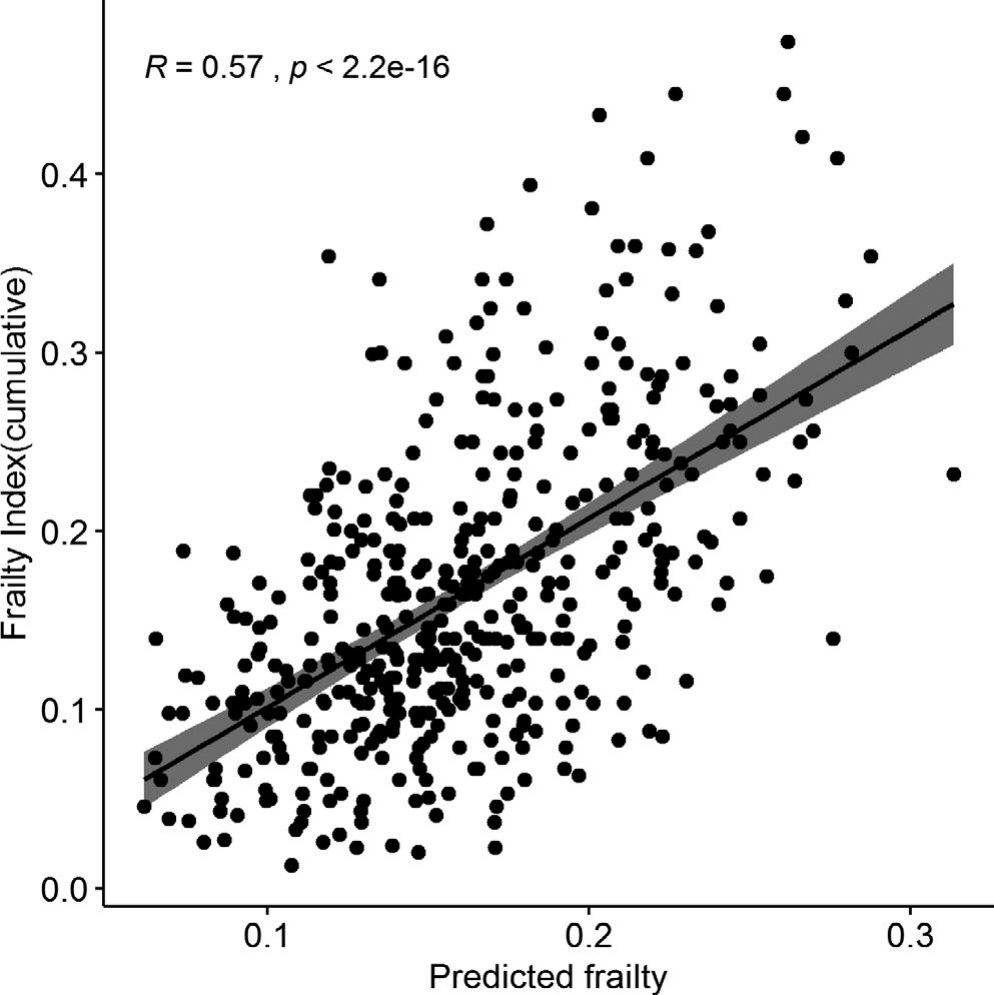
Correlation of observed cumulative frailty index and predicted frailty index using proteomic data. Frailty prediction using Elastic net regression method in 440 participants in the validation set. Correlation of predicted frailty using proteomic markers and cumulative frailty index was 0.57

We generated a proteomic signature of frailty using an elastic net regression by fitting in a cluster or subset of proteins from 4265 proteins that best predicted frailty. For this purpose, we divided our cohort into training and validation sets, with each group consisting of 440 unique participants. Elastic net regression applied to the training set selected 110 proteins out of the total number for proteomic frailty predictors (Table S5). Of these, 24 were associated with the frailty phenotype in the analysis above. The correlation between the predicted FI and observed cumulative FI in the validation cohort was *r* = 0.57 (*p* < 2.2 × 10^−16^) (Figure 2). In our prediction model, the correlation between predicted FI and cumulative FI did not differ by sex, and both fall in line with overall correlation of 0.57.

**Figure 3:**
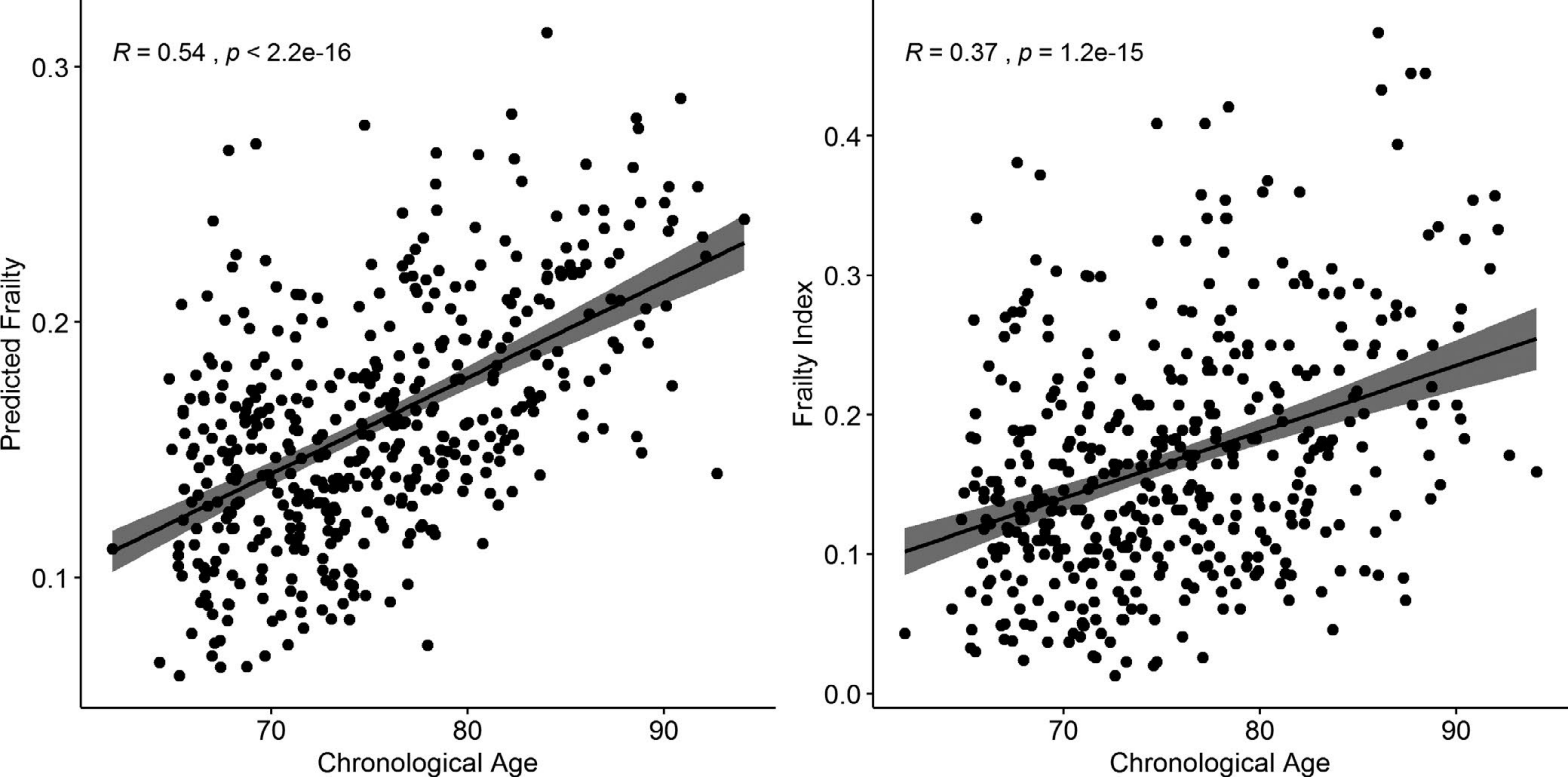
Correlation of observed cumulative frailty index and predicted frailty index with chronological Age. Higher correlation was observed with the predicted frailty and chronological age compared to actual cumulative frailty index and chronological age

Further, we analyzed the correlation of observed cumulative FI and predicted proteomic FI with chronological age. Interestingly, we found a higher correlation between chronological age and predicted proteomic FI (*r* = 0.54, *p* < 2.2 × 10^−16^) compared to observed FI (*r* = 0.37, *p* = 1.2 × 10^−15^) (Figure 3).

Association analysis results for all the 4265 SOMAmers with frailty‐ as well as gender‐based stratified results are provided in Tables S6‐S8.

## 3 DISCUSSION

The present study aimed to decipher the proteomic signature of frailty. To our knowledge, this is the first large‐scale proteomic study using the SomaScan Platform approach to elucidate the molecular phenotype of frailty at the proteomic level. The results of untargeted proteomic approach offer new insights into the pathogenesis and biomarkers of frailty.

The study identified a number of proteins that were positively as well as negatively associated with the clinical frailty phenotype. Top hit proteins that were positively associated with frailty (FABP, FABPA, and leptin) pointed toward a role for the lipid metabolism pathway in frailty. This was also confirmed by the pathway analysis as well as post hoc analysis including markers of lipid metabolism. Interestingly, the top two identified proteins belonged to the fatty acid‐binding protein family (~15 kDa proteins), which binds a hydrophobic ligand (fatty acids) in a reversible and noncovalent manner. Higher percentage of saturated fatty acids intake have been shown to be associated with higher levels of frailty (Jayanama, Theou, Godin, Cahill, & Rockwood, 2019). The top hit FABP, also referred to as FABP‐H, is a protein coded by *FABP3* gene (Chr 1p32‐1p33). It is expressed mainly in the heart and skeletal muscle and is involved in intracellular long‐chain fatty acid transport similar to other fatty acid‐binding proteins. H‐FABP has been shown to be a highly sensitive biomarker for acute coronary syndrome, including myocardial infarction, and it also predicts mortality after such an event (Kilcullen et al., 2007). The second top most hit, fatty acid‐binding protein adipocyte (FABPA), is coded by *FABP4* (chr 8q21.13) gene and is expressed mainly in adipocytes and macrophages. FABPA is closely linked with obesity and metabolic syndrome(Xu et al., 2006). It is associated with lipolysis and also acts as an adipokine playing a causative role in insulin resistance and atherosclerosis. Studies have also shown that Fabp‐deficient mice are protected against metabolic diseases, and have extended health span with protection against glucose intolerance and insulin resistance (Charles et al., 2017). These mice were also protected against inflammation and loss of adipose tissue integrity (Charles et al., 2017). Additionally, molecular inhibition of FABPA was found to be a successful therapeutic intervention against atherosclerosis and diabetes mellitus type 2 in a mouse model (Furuhashi et al., 2007). Leptin was the third top most protein to be positively associated with frailty. Leptin is a hormone produced mainly by the adipose cells and is involved in the regulation of body fat. Hence, it plays an important role in maintaining body weight and energy balance (Havel, 2000). However, this balance is lost in conditions such as obesity, which manifests as leptin resistance, a state mimicking insulin resistance in diabetes. Higher leptin levels are positively correlated with obesity and cardiovascular disease (Ekmen, Helvaci, Gunaldi, Sasani, & Yildirmak, 2016). Obesity has been associated with frailty (Blaum, Xue, Michelon, Semba, & Fried, 2005), and earlier studies also have shown higher levels of leptin to be associated with frailty (Lana, Valdés‐Bécares, Buño, Rodríguez‐Artalejo, & Lopez‐Garcia, 2017). Our post hoc analysis showed loss of significance of leptin as well as lowering of effect of fatty acid‐binding proteins with frailty when BMI was added to the model. This exploratory analysis points toward the possibility that the role of leptin in frailty pathogenesis may be mediated through obesity‐associated pathways. Further exploration of the lipid metabolism pathways in the context of frailty is needed to build on our findings. Interestingly, there has been a positive correlation between FABPA and leptin in disease conditions such as atherosclerotic plaque rupture (Lee et al., 2013). Other proteins positively associated with frailty in our study were coagulation factor IXab, IL‐1Ra, and FSTL3 that play important roles in homeostasis (Mari, Coppola, & Provenzano, 2008), inflammation (De Martinis, Franceschi, Monti, & Ginaldi, 2006), and cardiovascular outcomes (Heidecker et al., 2015), respectively, all important contributors to frailty.

The top most protein negatively associated with frailty was ANTR2 (anthrax toxin receptor protein 2), also called capillary morphogenesis gene 2 (CMG2), which is characterized by its binding ability to anthrax toxin. It is involved in angiogenesis and matrix assembly in the basement membrane. The association of ANTR2 with frailty is novel. But there are a number of previous studies pointing toward its possible role in frailty. Recent genetic studies have implicated *ANTXR2* gene coding for ANTR2 protein associated with hypertension (Park et al., 2014) as well as grip strength (Tikkanen et al., 2018). Grip strength is an important component of the frailty definition and hypertension is a known risk factor for frailty (Fried et al., 2001). However, further studies are warranted to determine ANTR2's mechanistic role in frailty. NELL1 was another top hit protein negatively associated with frailty. Another top hit protein negatively associated with frailty was NELL1, under expression of which has been associated with inadequate skeletal mineralization and age related osteoporosis. NELL‐1 improved bone mineral density in a rat model and bone formation in a sheep model (James et al., 2015).

Other well‐studied proteins previously associated with frailty and aging were also related to frailty in our study. These include higher levels of CRP and lower levels of KLOTHO (Shardell et al., 2017; Soysal et al., 2016). Recent studies, including those utilizing SomaScan array, have strengthened the role of MIC‐1/GDF‐15 protein, a stress‐induced cytokine from the TGF‐B family, with aging and associated traits (Tanaka et al., 2018; Wiklund et al., 2010). Increased expression of GDF‐15 has been implicated with aging (Tanaka et al., 2018) as well as mortality (Wiklund et al., 2010). Studies have shown up‐regulation of GDF‐15 in cardiovascular diseases (cardiomyopathies, heart failure, atrial fibrillation, and stroke) and with type 2 diabetes, where higher levels associated with fasting glucose, insulin resistance index, and glycated hemoglobin (Adela & Banerjee, 2015; Berezin, 2016).

Prior studies have demonstrated a higher prevalence of frailty among women compared to men (Fried et al., 2001). Women also have longer lifespans compared to men (Austad, 2006). We found a greater number of frailty‐associated proteins in females compared to males. These observations might suggest that there are more pathways leading to frailty in women compared to men. Frailty‐related proteins exclusive to males like thrombospondin‐2 (TSP‐2) play an important role in myocardial matrix integrity (Schroen et al., 2004). Increased expression of TSP‐2 predicted cardiac mortality in 992 elderly men even after adjustment for other cardiovascular risk factors (Golledge, Clancy, Hankey, & Norman, 2013). Expression of TSP‐2 rises in response to cardiac hypertrophy, which may lead to cardiac failure (Schroen et al., 2004). The greater number of female‐specific frailty‐associated proteins suggests the possibility of homeostasis disturbance that results in dysregulated protein networks to be more prevalent in females. These observations might be underlying basis for the observed "male–female health survival paradox," which is characterized by higher mortality rates in men despite higher rates of frailty and medical comorbidities in women (Kingston et al., 2014).

Our pathway analysis highlights the role of lipid metabolism as well as other pathway networks related to tissue development, skeletal and muscular system development and function in frailty. The different pathway analyses all aligned across the same top networks, suggesting a unifying biological model of frailty. Furthermore, these results offer up for consideration novel pathways involved in the pathogenesis of frailty, in addition to candidate pathways like inflammatory and oxidative stress response pathways.

We created a proteomic signature of frailty in our LonGenity cohort that achieved a correlation of 0.57 with actual frailty. A higher correlation between predicted and actual frailty may not have been observed due to the multifactorial nature of frailty, which is also influenced by factors such as age and gender. Better characterization of frailty by expanding the criterion as well as accounting for proteins that were not analyzed in the SomaScan array will help improve the concurrent validity of our biological frailty prediction model with physical frailty in the future. Predicted frailty (proteomic) was more strongly correlated with chronological age than actual frailty (FI) in the validation cohort. Hence, proteomic or biological models might become better predictors for frailty and chronological age. Further studies are warranted in this direction.

The current study has many strengths. We examined over 4000 proteins, making it one of the largest studies of proteomics of frailty to date, based on proteome and cohort sizes. Another strength of the study is the well‐characterized LonGenity cohort, which undergoes systematic clinical assessments and includes a validated and reliable cumulative deficit FI for capturing the multidimensional aspects of the frailty phenotype (Lehallier et al., 2019; Sathyan et al., 2018). Furthermore, this analysis included a relatively large number of subjects (*n* = 880) compared to previous proteomic studies in frailty. We acknowledge limitations. The SomaScan panel is not exhaustive, and unexamined proteins might contribute to frailty. The SomaScan is a relatively new and evolving technology that is continuously transforming with use of better aptamers as well as increase in number of proteins captured. Thus, repeating this study using future advanced techniques may yield even greater insights into the biology of frailty. The parent LonGenity study enrolled only Ashkenazi Jewish participants to maximize genetic discovery. Our cross‐sectional analysis does not shed light on the role of proteomics on the progression of frailty. Additional mechanistic studies and genetic studies in other diverse populations with longitudinal follow‐up are needed to validate the leading proteins and pathways identified in this study.

In conclusion, this study identified novel associations of proteins as well as pathways and frailty using the SomaScan array. This study also suggested the possibility of developing a better biological signature for frailty that can be defined by biomarkers. Future studies will need to investigate whether this proteomic signature can accurately identify and predict frailty in diverse populations. Further examination of the frailty‐associated proteins identified in this study may help develop potential interventions to mitigate frailty and to maintain functional independence in older adults.

## 4 MATERIALS AND METHODS

### 4.1 LonGenity cohort

The LonGenity study, established in 2007, is a cohort of AJ adults age 65 and older, who were either offspring of parents with exceptional longevity (OPEL), defined by having at least one parent who lived to age 95 and older, or offspring of parents with usual survival (OPUS), defined by having neither parent survive to age 95. The goal of the LonGenity study is to identify genotypes and phenotypes associated with longevity and successful aging. Study participants were systematically recruited using public records such as voter registration lists or through contacts at synagogues, community organizations, and advertisements in Jewish newspapers in the New York City area. Potential participants were contacted by telephone to assess interest and eligibility. Exclusion criteria include the following: presence of dementia using established cutscores of >8 on the Blessed Information‐Memory‐Concentration test and >2 on the AD8 at the initial screening interview, severe visual impairment, and having a sibling enrolled in the study. Eligible participants were invited to our research center for further evaluation. Participants received detailed medical history evaluation and cognitive testing at baseline as well as at annual follow‐up visits. All participants signed written informed consents for study assessment and genetic testing prior to enrollment. The Albert Einstein College of Medicine institutional review board approved the study protocol.

### 4.2 Frailty

The two most common approaches adopted to define frailty is either as a clinical syndrome (Fried et al., 2001) or as a cumulative deficit index (Rockwood & Mitnitski, 2007). In the present study, we used the cumulative deficit index proposed by Rockwood et al. (Searle et al., 2008) as it assesses a broader spectrum of disorders and conditions compared to the syndromic frailty definition. The cumulative deficit index also provides a continuous variable with meaningful quantification of frailty status independent of functional status or age (Kulminski et al., 2008). Phenotypic frailty proposed by Fried as clinical syndrome considered frailty as categorical variable with meaningful results restricted to non‐disabled older person only (Fried et al., 2001). The variables selected for the FI construction were based on standardized criteria that includes the following: association with health status, accumulates with age, biologically relevant, and must represent multiple organ systems (Searle et al., 2008). Further, variables should not saturate early with age like presbyopia, which are quite common by age 55 and are therefore excluded. A minimum of 30 variables is recommended for developing the FI (Rockwood & Mitnitski, 2007), which has been shown to predict deteriorating health status, institutionalization, and death (Rockwood & Mitnitski, 2007). Based on the recommended approach, 41 variables were included in the construction of the FI (Rockwood & Mitnitski, 2007). In case of binary variables, 0 represents no deficit and 1 represents a deficit. Continuous or rank variables were graded from 0 (no deficit) to 1 (maximum deficits). The variables and cutoffs used for construction of the FI are shown in Table S9. The FI was calculated by adding the number of deficits (value = 1) and dividing the sum by the total number of variables per participant, which resulted in a range of scores from 0 to 1 for each individual (Rockwood & Mitnitski, 2007). The FI showed a similar distribution to that obtained in earlier studies (Searle et al., 2008).

### 4.3 Proteomic assessment

Proteomics assessment was carried out using SomaScan assay from human plasma collected at baseline in LonGenity. Plasma samples were stored at −80°C, and 150 µl aliquots of plasma were sent to SomaLogic on dry ice. 5.0k SomaScan Assay includes 5284 SOMAmer reagents consisting of 5209 SOMAmer reagents that recognize human proteins with the remaining including 7 deprecated proteins, 12 hybridization control elution, 10 non‐biotin, 4 non‐cleavable, 22 non‐human proteins, and 20 spuriomers. SomaScan data standardization was carried out at SomaLogic, Inc., as previously described (Candia et al., 2017; Lehallier et al., 2019). It consisted of three steps—hybridization control normalization (HCN), median signal normalization (MSN), and calibration normalization (CN). HCN removed individual sample variance on the basis of signaling differences between micro array or Agilent scanner, whereas MSN removed inter‐sample variation within a plate arising from pipetting variation or other technical issues. CN removed variance across assay runs. Finally, median normalization to reference was performed on the quality control (QC), buffer, and individual samples. After implementing these QC checks, 960 sequences that failed QC check were removed. Further excluding spuriomers, non‐human proteins, non‐biotin, non‐cleavable, and deprecated markers, 4265 SOMAmer reagents were available for the analysis.

### 4.4 Statistical analysis

Baseline characteristics of participants were compared using descriptive statistics (Table 1). Relative fluorescence unit (RFU) values observed after data normalization procedures for each SOMAmer reagent were natural log transformed. Outliers were removed using median absolute deviation method. The primary objective of this study was to identify the association between proteins and frailty using a linear regression analysis. Analyses were adjusted for age, sex, and cohort status (OPUS‐OPEL). There are reports of higher frailty prevalence in females compared to males with age (Fried et al., 2001); therefore, we carried out a sex stratified analysis adjusting for age and cohort status. Multiple testing correction was carried out, and Bonferroni corrected *p*‐values of less than 1.17 × 10^−5^ (0.05/4265) were considered statistically significant.

### 4.5 Pathway analysis

Pathway analysis was conducted using frailty‐associated proteins to discover the biological pathways related to frailty. This was carried out using QIAGEN's Ingenuity^®^ Pathway Analysis (IPA^®^, QIAGEN Redwood City, www.qiagen.com/ingenuity) (Krämer, Green, Pollard, & Tugendreich, 2013). In this analysis, we included 143 proteins that were significantly associated with frailty in our initial analysis. IPA network analysis output consisted of a list of biological functions and sets of proteins, as well as scores (Score = − log10 (*p*‐value)) according to the fit of the set of proteins. Top networks were checked for concordance with pathway analysis using Reactome (www.reactome.org/) (Fabregat et al., 2017) as well as STRING (www.string‐db.org) (Szklarczyk et al., 2014).

We selected a biological (cholesterol) and clinical marker (BMI) linked to lipid metabolism to explore the relevance of our results. Both markers were significantly associated with frailty. BMI (*β* [*SE*] =0.0058 [0.0005]; *p* = 3.97 × 10^−26^) and cholesterol level (*β* [*SE*] = −0.0002 [0.00008]; *p* = 0.0063) were independently associated with frailty adjusting for age, gender, and cohort status. As a post hoc analysis, top frailty‐associated lipid metabolism pathway proteins (FABP, FABPA, and leptin) were reanalyzed for association with frailty using body mass index (BMI) and cholesterol level as covariates. Body mass index (BMI) was calculated according to the formula: BMI = weight in kg/(height in m)^2^. Lipid profiles including total cholesterol level were measured by standard automated method at the time of enrollment among participants at the Montefiore Medical Center clinical laboratories and the Biomarker Analytic Research Core at the Albert Einstein College of Medicine.

### 4.6 Frailty prediction using proteomic markers

Proteomic frailty predictor was constructed by utilizing a penalized regression model using the glmnet R package(Friedman, Hastie, & Tibshirani, 2010). Participants in the training set were selected using stratified random sampling method. Participants were selected from each of the 0.03 frailty score strata (0.00–0.03, 0.03–0.06, 0.06–0.09, 0.09–0.12, 0.12–0.15, 0.15–0.18, 0.18–0.21…). The remaining participants in the cohort contributed to the validation set. As a first step, frailty score was regressed on 4265 log‐transformed protein abundances. Using cv‐glmnet function, lambda value was selected on the basis of a 10‐fold cross‐validation using the training set. Program sets alpha value of 0.5 for elastic net regression.

## CONFLICT OF INTEREST

None declared.

## AUTHOR CONTRIBUTIONS

SS, NB, and JV contributed to the design of the study and interpretation of the data. SS, NB, SM, TG, and EA contributed to the acquisition of data and writing of the manuscript. EA and TG contributed to the analysis of the data. SS, EA, TG, SM, NB and JV contributed to the critical revisions of the manuscript. All the authors approved the final version of the manuscript and agree to be accountable for all aspects of the work.

## Supporting information

Figure S1

Table S1‐S4

Table S5‐S9

## Data Availability

Proteomic data used in this study are available upon request. Please contact the corresponding author for further information.
