# Supplementary figures and images for: Plasma proteomic profile of frailty
Source: Aging Cell. 2020 Aug 6;19(9):e13193. doi: 10.1111/acel.13193 (PMC7511877; doi:10.1111/acel.13193)

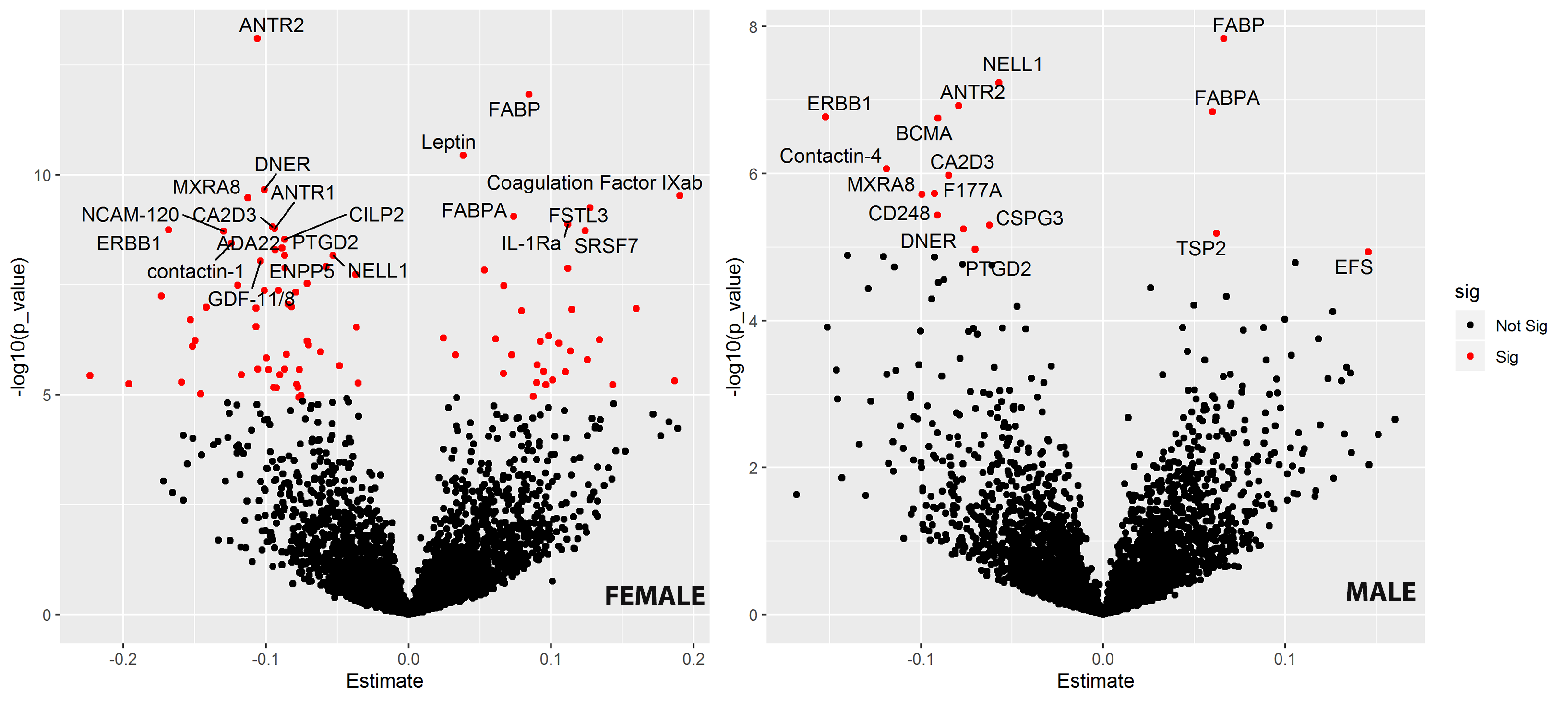

Supplement: Supplementary file 1 — Figure S1 [file ACEL-19-e13193-s001.tif]
