# Supplementary material for: Plasma proteomic profile of frailty
Source: Aging Cell. 2020 Aug 6;19(9):e13193. doi: 10.1111/acel.13193 (PMC7511877; doi:10.1111/acel.13193)
Supplement: Supplementary file 2 — Table S1‐S4 [file ACEL-19-e13193-s002.docx]

**Supplementary Table 1. 16 significant SOMAmer reagent targets associated with frailty phenotype in male participants.**

| **SeqId** | **SomaId** | UniProt | Target | Target Full Name | Estimate | Std Error | t value | p_value |
| --- | --- | --- | --- | --- | --- | --- | --- | --- |
| 5437-63 | SL001774 | P05413 | FABP | Fatty acid-binding protein, heart | 0.0662 | 0.0114 | 5.7910 | 1.45E-08 |
| 6544-33 | SL012542 | Q92832 | NELL1 | Protein kinase C-binding protein NELL1 | -0.0572 | 0.0103 | -5.5330 | 5.80E-08 |
| 15559-5 | SL011048 | P58335 | ANTR2 | Anthrax toxin receptor 2 | -0.0793 | 0.0147 | -5.3960 | 1.19E-07 |
| 15386-7 | SL005086 | P15090 | FABPA | Fatty acid-binding protein, adipocyte | 0.0600 | 0.0112 | 5.3620 | 1.43E-07 |
| 2677-1 | SL002644 | P00533 | ERBB1 | Epidermal growth factor receptor | -0.1525 | 0.0286 | -5.3270 | 1.70E-07 |
| 2665-26 | SL004672 | Q02223 | BCMA | Tumor necrosis factor receptor superfamily member 17 | -0.0907 | 0.0170 | -5.3210 | 1.77E-07 |
| 3298-52 | SL010454 | Q8IWV2 | Contactin-4 | Contactin-4 | -0.1190 | 0.0238 | -5.0030 | 8.63E-07 |
| 8885-6 | SL018710 | Q8IZS8 | CA2D3 | Voltage-dependent calcium channel subunit alpha-2/delta-3 | -0.0847 | 0.0171 | -4.9590 | 1.06E-06 |
| 8039-41 | SL018368 | Q8N128 | F177A | Protein FAM177A1 | -0.0925 | 0.0191 | -4.8440 | 1.85E-06 |
| 10521-10 | SL017989 | Q9BRK3 | MXRA8 | Matrix-remodeling-associated protein 8 | -0.0994 | 0.0205 | -4.8370 | 1.91E-06 |
| 15491-20 | SL008437 | Q9HCU0 | CD248 | Endosialin | -0.0910 | 0.0194 | -4.6990 | 3.65E-06 |
| 15573-110 | SL008782 | O14594 | CSPG3 | Neurocan core protein | -0.0623 | 0.0135 | -4.6290 | 5.03E-06 |
| 9769-48 | SL008968 | Q8NFT8 | DNER | Delta and Notch-like epidermal growth factor-related receptor | -0.0767 | 0.0167 | -4.6040 | 5.65E-06 |
| 3339-33 | SL007206 | P35442 | TSP2 | Thrombospondin-2 | 0.0621 | 0.0136 | 4.5760 | 6.45E-06 |
| 12549-33 | SL014636 | O60760 | PTGD2 | Hematopoietic prostaglandin D synthase | -0.0702 | 0.0157 | -4.4610 | 1.07E-05 |
| 12572-236 | SL019783 | O43281 | EFS | Embryonal Fyn-associated substrate | 0.1456 | 0.0328 | 4.4440 | 1.16E-05 |

16 proteins were associated with frailty in male participants.

**Supplementary Table 2. Top significant SOMAmer reagent targets associated with frailty phenotype in female participants.**

| **SeqId** | **SomaId** | **UniProt** | **Target** | **Target Full Name** | **Estimate** | **Std_Error** | **p_value** |
| --- | --- | --- | --- | --- | --- | --- | --- |
| 15559-5 | SL011048 | P58335 | ANTR2 | Anthrax toxin receptor 2 | -0.106 | 0.0138 | 7.87E-14 |
| 5437-63 | SL001774 | P05413 | FABP | Fatty acid-binding protein, heart | 0.0844 | 0.0116 | 1.47E-12 |
| 8484-24 | SL000498 | P41159 | Leptin | Leptin | 0.0383 | 0.0057 | 3.55E-11 |
| 9769-48 | SL008968 | Q8NFT8 | DNER | Delta and Notch-like epidermal growth factor-related receptor | -0.1012 | 0.0156 | 2.15E-10 |
| 5307-12 | SL004400 | P00740 | Coagulation Factor IXab | Coagulation factor IXab | 0.1903 | 0.0295 | 2.97E-10 |
| 10521-10 | SL017989 | Q9BRK3 | MXRA8 | Matrix-remodeling-associated protein 8 | -0.1127 | 0.0176 | 3.33E-10 |
| 3438-10 | SL009324 | O95633 | FSTL3 | Follistatin-related protein 3 | 0.1271 | 0.0201 | 5.59E-10 |
| 15386-7 | SL005086 | P15090 | FABPA | Fatty acid-binding protein, adipocyte | 0.0739 | 0.0118 | 8.73E-10 |
| 5353-89 | SL001990 | P18510 | IL-1Ra | Interleukin-1 receptor antagonist protein | 0.1116 | 0.018 | 1.31E-09 |
| 8885-6 | SL018710 | Q8IZS8 | CA2D3 | Voltage-dependent calcium channel subunit alpha-2/delta-3 | -0.0953 | 0.0155 | 1.50E-09 |
| 10464-6 | SL008696 | Q9H6X2 | ANTR1 | Anthrax toxin receptor 1 | -0.094 | 0.0153 | 1.67E-09 |
| 2677-1 | SL002644 | P00533 | ERBB1 | Epidermal growth factor receptor | -0.1683 | 0.0274 | 1.78E-09 |
| 12987-12 | SL019472 | Q16629 | SRSF7 | Serine/arginine-rich splicing factor 7 | 0.1238 | 0.0202 | 1.87E-09 |
| 4498-62 | SL003764 | P13591 | NCAM-120 | Neural cell adhesion molecule 1, 120 kDa isoform | -0.1298 | 0.0212 | 1.91E-09 |
| 8841-65 | SL008847 | Q8IUL8 | CILP2 | Cartilage intermediate layer protein 2 | -0.087 | 0.0144 | 2.92E-09 |
| 2974-61 | SL004855 | Q12860 | contactin-1 | Contactin-1 | -0.1243 | 0.0207 | 3.57E-09 |
| 12549-33 | SL014636 | O60760 | PTGD2 | Hematopoietic prostaglandin D synthase | -0.0888 | 0.0149 | 4.54E-09 |
| 7933-75 | SL018350 | Q9P0K1 | ADA22 | Disintegrin and metalloproteinase domain-containing protein 22 | -0.0935 | 0.0157 | 5.07E-09 |
| 6556-5 | SL012863 | Q9UJA9 | ENPP5 | Ectonucleotide pyrophosphatase/phosphodiesterase family member 5 | -0.0869 | 0.0147 | 6.72E-09 |
| 6544-33 | SL012542 | Q92832 | NELL1 | Protein kinase C-binding protein NELL1 | -0.0529 | 0.009 | 6.80E-09 |
| 2765-4 | SL021043 | O95390 O14793 | GDF-11/8 | Growth/differentiation factor 11/8 | -0.1038 | 0.0177 | 9.12E-09 |
| 5843-60 | SL003542 | Q96KQ7 | NG36 | Histone-lysine N-methyltransferase EHMT2 | -0.0578 | 0.01 | 1.21E-08 |
| 3364-76 | SL006910 | O60911 | Cathepsin V | Cathepsin L2 | -0.0866 | 0.015 | 1.31E-08 |
| 12574-36 | SL000405 | P20800 | Endothelin 2 | Endothelin-2 | 0.1116 | 0.0193 | 1.34E-08 |
| 7211-2 | SL005355 | P07998 | RNase 1 | Ribonuclease pancreatic | 0.0531 | 0.0092 | 1.46E-08 |
| 4187-49 | SL000247 | P52209 | 6-Phosphogluconate dehydrogenase | 6-phosphogluconate dehydrogenase, decarboxylating | -0.0371 | 0.0065 | 1.84E-08 |
| 15573-110 | SL008782 | O14594 | CSPG3 | Neurocan core protein | -0.0712 | 0.0126 | 2.91E-08 |
| 4880-21 | SL007756 | Q9UK05 | GDF2 | Growth/differentiation factor 2 | -0.1196 | 0.0213 | 3.22E-08 |
| 13463-1 | SL007573 | Q92626 | PXDN | Peroxidasin homolog | 0.0667 | 0.0118 | 3.28E-08 |
| 7218-87 | SL018093 | P14415 | AT1B2 | Sodium/potassium-transporting ATPase subunit beta-2 | -0.0910581 | 0.0163369 | 4.20E-08 |

88 proteins were associated with frailty in female participants

**Supplementary Table 3. Top IPA Bio functions and disease pathway associated with frailty**

| **Top Disease and Bio Functions** | | |
| --- | --- | --- |
| **Molecular and Cellular Functions** | | |
| **Name** | **p-value range** | **# Molecules** |
| Lipid Metabolism | 5.43E-04 - 9.95E-10 | 29 |
| Small Molecule Biochemistry | 5.59E-04 - 9.95E-10 | 37 |
| Molecular Transport | 5.59E-04 - 1.54E-08 | 41 |
| Cellular Movement | 6.28E-04 - 1.85E-08 | 44 |
| Cell-To-Cell Signaling and Interaction | 6.22E-04 - 2.58E-08 | 49 |
|  |  |  |
| **Physiological System Development and Function** | | |
| **Name** | **p-value range** | **# Molecules** |
| Tissue Development | 6.48E-04 - 3.85E-10 | 66 |
| Immune Cell Trafficking | 6.28E-04 - 1.85E-08 | 30 |
| Organismal Survival | 1.23E-04 - 2.05E-08 | 53 |
| Embryonic Development | 6.19E-04 - 1.33E-07 | 42 |
| Organismal Development | 6.48E-04 - 1.33E-07 | 77 |
|  |  |  |
| **Diseases and Disorders** | | |
| **Name** | **p-value range** | **# Molecules** |
| Cancer | 6.60E-04 - 8.39E-12 | 141 |
| Organismal Injury and Abnormalities | 6.60E-04 - 8.39E-12 | 143 |
| Gastrointestinal Disease | 6.11E-04 - 3.33E-09 | 131 |
| Dermatological Diseases and Conditions | 4.07E-04 - 1.17E-08 | 96 |
| Cardiovascular Disease | 4.76E-04 - 2.03E-08 | 43 |

**Supplementary Table 4. IPA generated networks ordered by a score denoting significance.**

| Top Diseases and Functions | Score | Focus Molecules | Molecules in Network |
| --- | --- | --- | --- |
| Organ Morphology, Skeletal and Muscular System Development and Function, Cell Morphology | 48 | 24 | ABCC6,ADAM22,ADAM23,APLP1,B4GALT6,C/EBP,CNTN1,CNTN4,CNTN5,DNER,ERBB,ERK1/2,FSTL3,GDF11,GDF15,GDF2,Growth hormone,Hif1,L1CAM,MSTN,NCAN,NDST1,NEO1, Notch,OMG,PTGFRN,RBBP9,RTN4R,Rsk,Secretase gamma,Smad,Smad1/5/8,Smad2/3,TFF3,WFIKKN2 |
| Tissue Development, Cell-To-Cell Signaling and Interaction, Cellular Assembly and Organization | 35 | 19 | ADAMTSL2,ANTXR1,ANTXR2,ARFIP2,ATP1B2,Akt,COL6A3,CRP,Collagen Alpha1,Collagen type IV, Collagen type VI, Cyclin A,FAIM,FBLN1,Fibrinogen,GSR,IMPAD1,ITGAV,ITGB5,Integrin alpha V beta 3,JPT1,KL,Laminin (complex), Laminin1,MFAP5,NADPH oxidase,NELL1,PGD,PXDN,Pdgf Ab,Rap1,TSH,Thrombospondin,collagen type i (family),elastase |
| Hematological Disease, Hereditary Disorder, Organismal Injury and Abnormalities | 35 | 19 | Alpha catenin, BOC, CTSV, CaMKII, Calcineurin protein(s),Collagen type I (complex),Collagen(s),F9,FAP,GLRX3, GPC3,HHIP,HTRA1,Hedgehog,LMAN2,MAP2K1/2,Mmp,NCAM1,NCAM2,NFkB (complex),NLRP4,OAF,PDGF BB,PP2A,Pdgf (complex),SERPINC1,SRSF7,STAT5a/b, Serine Protease,THBS2,TNXB,TRA2B,VOPP1,collagen,trypsin |
| Lipid Metabolism, Molecular Transport, Small Molecule Biochemistry | 35 | 19 | ALB,AMBP,APOC1,APOM,BCAN,CST3,Dynamin,EGFR,FABP4,GOT,Gap,HDL,HDL-cholesterol,IL12 (complex),IL1RN,INHBC,ITIH5,LEP,LILRA4,LRIG3,Ldh (complex),MHC Class II (complex),Mucin,Nos,PI3K (family),PRKAA, PTPRD, Pro-inflammatory Cytokine,Proinsulin,RIDA,SLITRK3,STX12,STX1A,Sod,Syntaxin |
| Cellular Development, Cellular Growth and Proliferation, Hematological System Development and Function | 26 | 15 | ABCC5,ATOX1,B4GALT2,CA6,CDK5,CHST1,DCLK3,Eda,FAM20B,HNF4A,HS6ST3,IL4,ISLR2,KLK1,LILRA4,MAFG,NTRK1,NUPR1,OSM,PEPD,PMM2,PPP1CA,RBM23,RBP5,RNASE4,SEMG1,SPOCK2,STAT,SULT1A1,TMEM132D,TNF,TRIM25,beta-estradiol,ribose,sulfotransferase |
| Cellular Assembly and Organization, Cellular Movement, Nervous System Development and Function | 21 | 13 | 26s Proteasome,ARFIP2,Actin,BHLHE22,Beta Arrestin,CACNA1I,CACNA2D3,CAP2,CDH12,COL4A6,Cacna2d,Calmodulin,EF-1 alpha,EHMT2,EWSR1,GIP,GSN,Hdac,Histone h3,Insulin,PPP1R1A,R-type Calcium Channel, RNA polymerase II,RNASE1,RNASE4,ROBO2,Rac,SPAG7,SUMO2,TNNT2,ZHX3,caspase,estrogen receptor, myosin-light-chain kinase, voltage-gated calcium channel |

The highest-scoring network, which comprises 24 proteins in our list, revealed significant changes in Organ Morphology, Skeletal and Muscular System Development and Function and Cell Morphology.
